# Supplementary material for: Invertebrates of Siberia, a Potential Source of Animal Protein for Innovative Food and Feed Production: Biomass Nutrient Composition Change in the Earthworm Eisenia fetida (Savigny, 1826) and the House Cricket Acheta domesticus (Linnaeus, 1758)
Source: Insects. 2025 Jun 16;16(6):632. doi: 10.3390/insects16060632 (PMC12193537; doi:10.3390/insects16060632)
Supplement: Supplementary file 1 [file insects-16-00632-s001.zip › insects-3656120-supplementary.pdf]

Invertebrates of Siberia, a potential source of animal protein for innovative human food production. 7. Biomass nutrient composition change in worms and crickets

S.E. Tshernyshev, Babenko A.S., R.T-O. Baghirov, V.P. Modyaeva, M.D. Morozova, K.E. Skriptcova, E.Yu. Subbotina, M.V. Shcherbakov, A.V. Simakova

Table S1. **Control group**

Index of content of the nutrient detected	Sample No.319A *Acheta domesticus* (Linnaeus, 1758)	Sample No.293 *Acheta domesticus* (Linnaeus, 1758)	Sample No.314A *Eisenia fetida* (Bouché, 1972)	Sample No.292 *Eisenia fetida* (Bouché, 1972)	Method applied (GOST number)	Human daily requirement according to
Dose	1×	2×	1×	2×		
Vitamins						
B1 (thiamine), mg/100 g	0.033±0.002	0.052±0.003	1.223±0.061	1.3±0.1	EN 14122	0.3–1.5 mg/day
B2 (riboflavin), mg/100 g	3.226±0.161	3.8±0.2	9.369±0.468	11.7±0.6	EN 14152	1.8 mg/day
B3 (niacinamide), mg/100 g	3.292±0.165	4.2±0.2	22.347±1.117	17.5±0.9	31483	20 mg/day
B6 (pyridoxine), mg/100 g	0.127±0.006	0.19±0.02	0.101±0.005	0.15±0.01	EN 14164	2.0 mg/day
B7 (biotin), mg/100 g	0.011±0.001		0.022±0.001		P 50929	
B9 (folic acid), µg/100 g	0.122±0.006	0.15±0.01	0.029±0.001	0.042±0.002	31483	400 µg/100 g
B12 (cyanocobalamin), µg /100 g	0.282±0.014	0.33±0.05	0.026±0.001	0.019±0.001	ISO 20634	0.3–3.0 µg/100 g
E (α-tocopherol), mg/100 g	2.083±0.104	2.2±0.1	3.773±0.189	5.3±0.3	32307	15 µg/100 g
A (retinol palmitate), µg/100 g	4.752±0.238	5.2±0.3	0.799±0.040	0.85±0.04	32307	400–1000 µg/100 g

D3 (cholecalciferol), µg/100 g	0.058±0.003	0.075±0.004	0.026±0.001	0.033±0.002	32307	
K (fillokinone), µg/100 g	6.558±0.328	7.4±0.4	7.765±0.388	8.8±0.4	EN 14148	
C (ascorbic acid), mg/100 g	2.908±0.145	2.6±0.1	3.773±0.189	5.0±0.2	34151	
Minerals						
Fe, iron, mg/100 g	0.541±0.081	2.3±0.6	24.6±3.7	12.1±0.6	ICP MS	4–18 mg/day
Se, selenium, µg/100 g	0.099±0.015	0.10±0.02	0.37±0.06	0.09±0.02	ICP MS	10–70 µg/100 g
Zn, zinc, mg/100 g	15.9±2.4	6.8±0.5	7.0±1.1	3.3±1.1	ICP MS	3–12 mg/day
Mn, manganese, mg/100 g	3.12±047	1.67±0.03	1.1±0.2	0.74±0.03	ICP MS	2 mg/day
Cu, copper, mg/100 g	2.7±0.4	0.78±0.12	0.52±0.08	0.23±0.02	ICP MS	0.5–1.0 mg/day
Mg, magnesium, mg/100 g	92.0±13.8	45.3±2.3	71.3±10.7	28.7±1.4	ICP MS 32009	55–400 mg/day
P, phosphorus, mg/100 g	791.3±78.7	314.7±15.5	495.7±74.4	159.6±7.4	ICP MS 32009	300–1200 mg/day
Pb, lead, mg/100 g	0.002		0.038±0.004		ICP MS 32009	

Hg, mercury, mg/100 g	0.0004		0.0008		ICP MS 32009	
Mo, molybdenum, mg/100 g	0.070	0.047±0.005	0.034	0.030	ICP MS 32009	
I, iodine, mg/100 g	0.062	1.1±0.2	0.024	5.4	ICP MS 32009	
Ca, calcium, mg/100 g	90.6±13.2	74.8±3.7	147.7±22.2	82.9±4.1	ICP MS 32009	
Na, sodium, mg/100 g	355.4±53.3	232.2±12.3	268.9±40.3	196.3±9.3	ICP MS 32009	
K, potassium, mg/100 g	1089.9±163.5	455.1±22.5	874.5±131.2	229.1±11.2	ICP MS 32009	
Cl, chlorine, mg/100 g	934.5±140.1	661.5±33.7	153.6±23.0	615.5±30.7	ICP MS 32009	
Other nutrients mass fraction, %						
Ash content	1.44±0.134	1.20±0.12	0.40±0.04	0.72±0.04	27494	
Fat	2.03±0.20	3.44±0.17	0.27±0.03	0.35±0.03	23042	70 до 154 g/day
Protein	16.9±1.7	16.2±0.8	17.6±1.7	16.6±0.7	25011	65–117 g/day with 60% animal protein
Carbohydrate	35.0±3.5	28.0±1.4	10.6±1.1	8.7±0.4	32167	170–420 g/day

Chitin	4.05±0.40	4.8±0.2	0.08±0.01	1.08±0.01	7636	20 g/day
Cellulose		12.55±0.65		0.96±0.11	31675	
Water		35.4±1.7		61.4	13586.5	
Caloricity, kcal	226.4	207	115.2	104.4	Calculation method	individually

Table S2. **Biotin, vitamin C**

Index of content of the nutrient detected	Sample No.320A *Acheta domesticus* (Linnaeus, 1758)	Sample No.295 *Acheta domesticus* (Linnaeus, 1758)	Sample No.315A *Eisenia fetida* (Bouché, 1972)	Sample No.294 *Eisenia fetida* (Bouché, 1972)	Method applied (GOST number)	Human daily requirement according to
Dose	1×	2×	1×	2×		
Vitamins						
B1 (thiamine), mg/100 g	0,045±0,002		1,247±0,062		EN 14122	0.3–1.5 mg/day
B2 (riboflavin), mg/100 g	3,38±0,167		17,521±0,876		EN 14152	1.8 mg/day
B3 (niacinamide), mg/100 g	3,441±0,172		39,143±1,951		31483	20 mg/day
B6 (pyridoxine), mg/100 g	0,234±0,012		0,222±0,011		EN 14164	2,0 mg/day
B7 (biotin), mg/100 g	0,020±0,001		0,042±0,002		P 50929	
B9 (folic acid), µg/100 g	0,119±0,006		0,059±0,003		31483	400 µg/100 g
B12 (cyanocobalamin), µg /100 g	0,553±0,028		0,052±0,003		ISO 20634	0.3–3.0 µg/100 g
E (α-tocopherol), mg/100 g	2,330±0,116		4,572±0,229		32307	15 µg/100 g
A (retinol palmitate), µg/100 g	5,995±0,300		1,696±0,086		32307	400–1000 µg/100 g
D3 (cholecalciferol), µg/100 g	0,073±0,004		0,052±0,003		32307	

K (fillokinone), µg/100 g	7,766±0,0388		2,418±0,121		EN 14148	
C (ascorbic acid), mg/100 g	2,398±0,120		6,213±0,311		34151	
Minerals						
Fe, iron, mg/100 g	0,720±0,110		26,4±4,3		ICP MS	4–18 mg/day
Se, selenium, µg/100 g	0,078±0,012		0,41±0,05		ICP MS	10–70 µg/100 g
Zn, zinc, mg/100 g	20,6±3,1		9,0±1,1		ICP MS	3–12 mg/day
Mn, manganese, mg/100 g	3,5±0,5		1,5±0,2		ICP MS	2 mg/day
Cu, copper, mg/100 g	2,55±0,38		0,47±0,05		ICP MS	0.5–1.0 mg/day
Mg, magnesium, mg/100 g	122,6±18,4		70,6±10,7		ICP MS 32009	55–400 mg/day
P, phosphorus, mg/100 g	908,7±136,3		566,5±83,0		ICP MS 32009	300–1200 mg/day
Pb, lead, mg/100 g	0,004		0,018±0,003		ICP MS 32009	
Hg, mercury, mg/100 g	0,0002		0,0005		ICP MS 32009	

Mo, molybdenum, mg/100 g	0,081		0,035		ICP MS 32009	
I, iodine, mg/100 g	0,046		0,0085		ICP MS 32009	
Ca, calcium, mg/100 g	108,2±16,2		142,5±21,0		ICP MS 32009	
Na, sodium, mg/100 g	352,9±52,9		289,7±45,3		ICP MS 32009	
K, potassium, mg/100 g	1126,4±169,0		844,6±115,2		ICP MS 32009	
Cl, chlorine, mg/100 g	615,1±92,3		66,2±11,4		ICP MS 32009	
Other nutrients mass fraction, %						
Ash content	1,48±0,14	1,30±0,04	0,64±0,06	0,72±0,04	27494	
Fat	2,59±0,26	5,12±0,05	0,23±0,02	0,32±0,03	23042	70 до 154 g/day
Protein	17,1±1,7	16,8±0,8	21,6±2,1	18,5±0,7	25011	65–117 g/day with 60% animal protein
Carbohydrate	29,9±3,0	28,4±0,4	9,1±0,9	8,9±0,4	32167	170–420 g/day
Chitin	9,98±0,90	5,14±0,01	0,06±0,01	1,28±0,01	7636	20 g/day

Cellulose		13,88±0,65		0,97±0,11	31675	
Water		32,2±1,6		67,4	13586.5	
Caloricity, kcal	211,3	227	124,8	112,9	Calculation method	individuall y

Table S3. **B-group vitamins**

Index of content of the nutrient detected	Sample No.322A *Acheta domesticus* (Linnaeus, 1758)	Sample No.299 *Acheta domesticus* (Linnaeus, 1758)	Sample No.317A *Eisenia fetida* (Bouché, 1972)	Sample No.298 *Eisenia fetida* (Bouché, 1972)	Method applied (GOST number)	Human daily requirement according to
Dose	1×	2×	1×	2×		
Vitamins						
B1 (thiamine), mg/100 g	0,032±0,002	0,055±0,003	1,182±0,059	1,4±0,1	EN 14122	0.3–1.5 mg/day
B2 (riboflavin), mg/100 g	2,879±0,144	4,7±0,2	13,610±0,680	13,2±0,5	EN 14152	1.8 mg/day
B3 (niacinamide), mg/100 g	4,445±0,222	6,9±0,3	31,527±1,576	23,3±1,1	31483	20 mg/day
B6 (pyridoxine), mg/100 g	0,202±0,010	0,48±0,03	0,201±0,010	0,27±0,02	EN 14164	2,0 mg/day
B7 (biotin), mg/100 g	0,018±0,001		0,038±0,003		P 50929	
B9 (folic acid), (mg)/100 g	0,134±0,007	0,38±0,02	0,040±0,002	0,048±0,02	31483	400 µg/100 g
B12 (cyanocobalamin), µg /100 g	0,554±0,028	0,56±0,004	0,050±0,002	0,036±0,003	ISO 20634	0.3–3.0 µg/100 g
E (α-tocopherol), mg/100 g	1,877±0,094		6,252±0,313		32307	15 µg/100 g
A (retinol palmitate), µg/100 g	6,625±0,331		1,633±0,082		32307	400–1000 µg/100 g
D3 (cholecalciferol), µg/100 g	0,077±0,004		0,043±0,002		32307	
K (fillokinone), µg/100 g	7,205±0,360		2,220±0,111		EN 14148	
C (ascorbic acid), mg/100 g	2,669±0,133		5,361±0,268		34151	

Minerals						
Fe, iron, mg/100 g	0,34±0,03		25,7±3,2		ICP MS	4–18 mg/day
Se, selenium, µg/100 g	0,051±0,005		0,042±0,006		ICP MS	10–70 µg/100 g
Zn, zinc, mg/100 g	8,5±0,8		7,2±0,8		ICP MS	3–12 mg/day
Mn, manganese, mg/100 g	2,1±0,2		1,5±0,2		ICP MS	2 mg/day
Cu, copper, mg/100 g	1,72±0,17		0,55±0,08		ICP MS	0.5–1.0 mg/day
Mg, magnesium, mg/100 g	105,3±10,5		67,3±8,2		ICP MS 32009	55–400 mg/day
P, phosphorus, mg/100 g	857,5±86±7		605,0±93,0		ICP MS 32009	300–1200 mg/day
Pb, lead, mg/100 g	0,0026		0,006±0,001		ICP MS 32009	
Hg, mercury, mg/100 g	0,0006		0,0003		ICP MS 32009	
Mo, molybdenum, mg/100 g	0,0023		0,033		ICP MS 32009	
I, iodine, µg/100 g	0,026		0,0069		ICP MS 32009	

Ca, calcium, mg/100 g	112,2±11,2		128,3±21,0		ICP MS 32009	
Na, sodium, mg/100 g	311,5±31,1		352,2±51,2		ICP MS 32009	
K, potassium, mg/100 g	1080,6±108,0		783,5±111,0		ICP MS 32009	
Cl, chlorine, mg/100 g	487,5±48,7		31,4±3,2		ICP MS 32009	
Other nutrients mass fraction, %						
Ash content	1,60±0,16	1,17±0,11	0,62±0,06	1,12±0,11	27494	
Fat	2,27±0,22	5,52±0,07	0,34±0,03	0,33±0,07	23042	70 до 154 g/day
Protein	16,6±1,7	21,3±0,8	17,2±1,7	17,9±0,8	25011	65–117 g/day with 60% animal protein
Carbohydrate	34,1±3,4	28,7±0,8	8,0±0,8	8,8±0,8	32167	170–420 g/day
Chitin	4,03±0,40	4,8±0,2	0,07±0,02	1,12±0,05	7636	20 g/day
Cellulose		12,1±0,6		0,90±0,09	31675	
Water		37,3±1,5		63,3±3,3	13586.5	

Caloricity, kcal	223,2	249,7	103,9	109,8	Calculation method	individually

Table S4. **Fat soluble vitamins**

Index of content of the nutrient detected	Sample No.323A *Acheta domesticus* (Linnaeus, 1758)	Sample No.301 *Acheta domesticus* (Linnaeus, 1758)	Sample No.318A *Eisenia fetida* (Bouché, 1972)	Sample No.27A *Eisenia fetida* (Bouché, 1972)	Method applied (GOST number)	Human daily requirement according to
Dose	1×	2×	1×	2×		
Vitamins						
B1 (thiamine), mg/100 g	0,048±0,002		1,231±0,062		EN 14122	0.3–1.5 mg/day
B2 (riboflavin), mg/100 g	3,546±0,177		12,472±0,624		EN 14152	1.8 mg/day
B3 (niacinamide), mg/100 g	3,860±0,193		43,541±2,177		31483	20 mg/day
B6 (pyridoxine), mg/100 g	0,205±0,010		0,205±0,010		EN 14164	2,0 mg/day
B7 (biotin), mg/100 g	0,017±0,001		0,033±0,002		P 50929	
B9 (folic acid), mg/100 g	0,152±0,008		0,048±0,002		31483	400 μg/100 g
B12 (cyanocobalamin), μg /100 g	0,510±0,025		0,053±0,003		ISO 20634	0.3–3.0 μg/100 g
E (α-tocopherol), mg/100 g	2,056±0,103	3,4±0,2	5,829±0,291	8,9±0,5	32307	15 μg/100 g
A (retinol palmitate), μg/100 g	6,995±0,380	7,7±0,3	1,260±0,063	2,24±0,11	32307	400–1000 μg/100 g
D3 (cholecalciferol), μg/100 g	0,084±0,004	0,088±0,004	0,047±0,002	0,048±0,003	32307	
K (fillokinone), μg/100 g	7,605±0,309	15,2±0,5	1,829±0,091	12,7±0,6	EN 14148	
C (ascorbic acid), mg/100 g	2,725±0,136	3,4±0,2	5,609±0,280	9,4±0,5	34151	

Minerals						
Fe, iron, mg/100 g	0,39±0,04		24,9±0,280		ICP MS	4–18 mg/day
Se, selenium, µg/100 g	0,062±0,006		0,035±0,006		ICP MS	10–70 µg/100 g
Zn, zinc, mg/100 g	11,5±0,9		6,9±0,7		ICP MS	3–12 mg/day
Mn, manganese, mg/100 g	3,06±0,28		1,2±0,2		ICP MS	2 mg/day
Cu, copper, mg/100 g	2,88±0,28		0,53±0,08		ICP MS	0.5–1.0 mg/day
Mg, magnesium, mg/100 g	98,3±9,8		69,8±10,2		ICP MS 32009	55–400 mg/day
P, phosphorus, mg/100 g	785,9±79,6		523,7±73,0		ICP MS 32009	300–1200 mg/day
Pb, lead, mg/100 g	0,0021		0,005±0,001		ICP MS 32009	
Hg, mercury, mg/100 g	0,0011		0,0006		ICP MS 32009	
Mo, molybdenum, mg/100 g	0,005		0,035		ICP MS 32009	
I, iodine, µg/100 g	0,062		0,005		ICP MS 32009	

Ca, calcium, mg/100 g	1002,4±100,2		142,5±22,0		ICP MS 32009	
Na, sodium, mg/100 g	333,8±33,3		315,6±41,5		ICP MS 32009	
K, potassium, mg/100 g	10,85,5±108,5		833,8±124,0		ICP MS 32009	
Cl, chlorine, mg/100 g	517,8±51,7		153,4±23,2		ICP MS 32009	
Other nutrients mass fraction, %						
Ash content	1,55±0,16	1,12±0,1	0,64±0,06	1,2±0,1	27494	
Fat	1,6±0,16	4,97±0,03	0,46±0,05	0,36±0,02	23042	70 до 154 g/day
Protein	17,1±1,7	17,2±0,5	18,2±1,8	17,8±0,*	25011	65–117 g/day with 60% animal protein
Carbohydrate	39,2±3,9	28,7±0,8	9,9±0,9	8,5±0,8	32167	170–420 g/day
Chitin	11,4±1,1	4,5±0,02	0,05±0,01	1,03±0,01	7636	20 g/day
Cellulose		15,0±0,2		0,90±0,09	31675	
Water		42,2±2,4		65,2±3,3	13586.5	

Caloricity, kcal	239,6	228,7	116,5	108,5	Calculation method	individually

Table S5. **Minerals**

Index of content of the nutrient detected	Sample No.321A *Acheta domesticus* (Linnaeus, 1758)	Sample No.296 *Acheta domesticus* (Linnaeus, 1758)	Sample No.316A *Eisenia fetida* (Bouché, 1972)	Sample No.27A *Eisenia fetida* (Bouché, 1972)	Method applied (GOST number)	Human daily requirement according to
Dose	1×	2×	1×	2×		
Vitamins						
B1 (thiamine), mg/100 g	0,038±0,002		1,631±0,082		EN 14122	0.3–1.5 mg/day
B2 (riboflavin), mg/100 g	3,215±0,161		13,360±0,668		EN 14152	1.8 mg/day
B3 (niacinamide), mg/100 g	4,363±0,218		41,717±2,086		31483	20 mg/day
B6 (pyridoxine), mg/100 g	0,224±0,011		0,176±0,009		EN 14164	2,0 mg/day
B7 (biotin), mg/100 g	0,020±0,001		0,031±0,002		P 50929	
B9 (folic acid), mg/100 g	0,159±0,008		0,060±0,003		31483	400 μg/100 g
B12 (cyanocobalamin), μg /100 g	0,556±0,028		0,058±0,003		ISO 20634	0.3–3.0 μg/100 g
E (α-tocopherol), mg/100 g	2,750±0,138		4,874±0,244		32307	15 μg/100 g
A (retinol palmitate), μg/100 g	7,119±0,356		1,487±0,074		32307	400–1000 μg/100 g
D3 (cholecalciferol), μg/100 g	0,057±0,003		0,042±0,002		32307	
K (fillokinone), μg/100 g	6,967±0,348		2,335±0,117		EN 14148	
C (ascorbic acid), mg/100 g	3,162±0,158		6,378±0,319		34151	

Minerals						
Fe, iron, mg/100 g	0,7±0,1	2,6±0,6	29,4±4,4	17,0±0,8	ICP MS	4–18 mg/day
Se, selenium, µg/100 g	0,078±0,012	0,09±0,02	0,055±0,008	0,05±0,01	ICP MS	10–70 µg/100 g
Zn, zinc, mg/100 g	20,6±3,1	4,3±0,2	9,8±1,3	3,98±0,24	ICP MS	3–12 mg/day
Mn, manganese, mg/100 g	3,5±0,5	0,96±0,03	1,8±0,3	1,27±0,06	ICP MS	2 mg/day
Cu, copper, mg/100 g	2,55±0,38	0,74±2,2	0,62±0,09	0,20±0,10	ICP MS	0.5–1.0 mg/day
Mg, magnesium, mg/100 g	122,6±18,4	40,7±2,2	73,9±11,1	40,2±2,2	ICP MS 32009	55–400 mg/day
P, phosphorus, mg/100 g	908,7±136,3	280,3±14,2	646,0±96,0	190,6±9,2	ICP MS 32009	300–1200 mg/day
Pb, lead, mg/100 g	0,0037		0,010±0,001		ICP MS 32009	
Hg, mercury, mg/100 g	0,0017		0,0005		ICP MS 32009	
Mo, molybdenum, mg/100 g	0,0081	0.044±0,005	0,032	0,025±0,005	ICP MS 32009	
I, iodine, mg/100 g	0,046	0,9±0,2	0,0088	4,4±0,2	ICP MS 32009	

Ca, calcium, mg/100 g	108,2±16,2	79,2±3,8	152,7±22,9	107,2±5,3	ICP MS 32009	
Na, sodium, mg/100 g	352,9±52,9	195,5±11,7	376,6±56,5	230,5±12,5	ICP MS 32009	
K, potassium, mg/100 g	1126,4±169,0	338,3±33,8	806,6±121,0	251,3±13,8	ICP MS 32009	
Cl, chlorine, mg/100 g	615,1±92,3	609±60	35,9±3,5	681,60	ICP MS 32009	
Other nutrients mass fraction, %						
Ash content	1,90±0,19	1,34±0,13	0,58±0,06	0,79±0,11	27494	
Fat	2,84±0,28	4,78±0,23	0,94±0,09	0,78±0,07	23042	70 до 154 g/day
Protein	17,3±1,7	16,9±0,8	17,8±1,7	17,3±0,8	25011	65–117 g/day with 60% animal protein
Carbohydrate	32,7±3,3	28,9±1,4	8,3±0,8	8,9±0,8	32167	170–420 g/day
Chitin	5,90±0,59	4,2±0,2	0,08±0,02	1,68±0,08	7636	20 g/day
Cellulose		14,72±0,77		0,97±0,10	31675	
Water		35,9±1,7		65,3±3,3	13586.5	

Caloricity, kcal	225,6	226	112,9	116	Calculation method	individually
